# 1804. A Hospital System Utilization of EMR towards New Jersey’s Goal of Viral Hepatitis and HIV Elimination

**DOI:** 10.1093/ofid/ofad500.1633

**Published:** 2023-11-27

**Authors:** Binghong Xu, Ruth P Brogden, Aryana Velez, Meenakumarie Pillay, Jaymie Yango, Ammie J Patel, Eric Handler, Christopher Crean, Mityanand Ramnarine, Lauren Trattner, Stephen O’Mahony, Su H Wang

**Affiliations:** Center for Asian Health, RWJBH-Saint Barnabas Medical Center, Florham Park, New Jersey; RWJBH-Saint Barnabas Medical Center, Florham Park, New Jersey; Cooperman Barnabas Medical Center, Florham Park, New Jersey; Cooperman Barnabas Medical Center, Florham Park, New Jersey; RWJBH - Saint Barnabas Medical Center, Livingston, New Jersey; Ernest Mario School of Pharmacy, Rutgers, the State University of New Jersey, Livingston, New Jersey; Cooperman Barnabas Medical Center, Florham Park, New Jersey; Somerset Medical Center, Somerville, New Jersey; Rahway Hospital, Rahway, New Jersey; Rahway Hospital, Rahway, New Jersey; RWJBarnabas Health, Livingston, New Jersey; Cooperman Barnabas Medical Center | Hepatitis B Foundation, Florham Park, New Jersey

## Abstract

**Background:**

New Jersey plans to reduce 75% of new HIV infections and promote access to testing and linkage-to-care (LTC) to end the HIV epidemic by 2025. In the US, CDC 2020 hepatitis surveillance report indicated cases of hepatitis C in 2020 corresponded to a 124% increase from 2013. Globally, if the HBV effort remains unchanged, the annual deaths from HBV are projected to increase by 39% from 2015 to 2030. We implemented an electronic medical record (EMR) based screening algorithm to scale up HBV, HCV, and HIV screening and provide LTC.

**Methods:**

In March 2018, the EMR was modified at Cooperman Barnabas Medical Center in the Emergency Department (ED) to start automated screening, we expanded the screening criteria to boost identifying the undiagnosed. Subsequently, the program expanded to Somerset Medical Center and Rahway Hospital. Patient Navigators receive a real-time alert of positive results to contact patients and arrange LTC, the multidisciplinary team offers evaluation and treatment.

**Figure 1.**
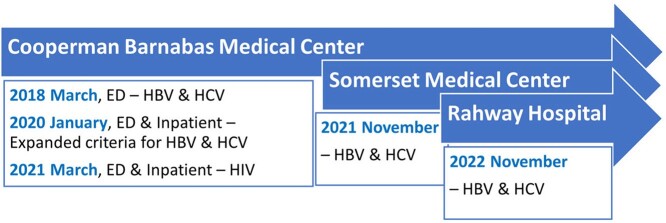
Expanding Automated Screening Program

**Results:**

We found HIV prevalence in CBMC is 1.5%, close to 5 times the NJ statewide 397.2 per 100,000 population. HBV infection rates varied in three hospitals, representative of the diverse population in their service areas. The HBV patients we confirmed came from more than 170 countries with different insurance statuses, the majority are immigrants from endemic countries or with high-risk behaviors identified. From Aug 2022, new HbsAg + cases reflex to HDVAb test, where 5% resulted HDVAb+ and no HDVRNA+. HCV prevalence rates are more consistent among the three sites, however, the percentages of the birth cohort (1945-1965) are varied. LTC rates are higher in HBV patients than HCV, thus far, 41 patients were cured of HCV from our team.

**Figure 2.**
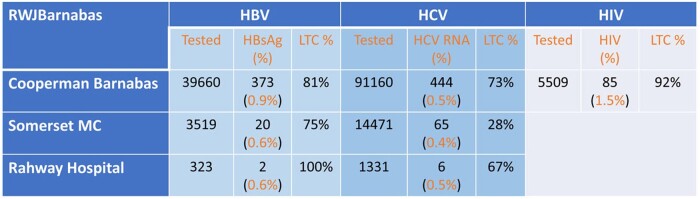
Total Numbers of HBV, HCV & HIV Tested, Prevalence & LTC

**Conclusion:**

EMR automated screening significantly scales up the diagnosis of HBV, HCV and HIV, as well as identifying HIV at-risk patients to receive comprehensive preventative services. HBV, HCV and HIV share similar risks, LTC is crucial to treat current infections and is beneficial to prevent transmission and potential exposure. In summary, automated screening and LTC work robustly toward elimination.

**Disclosures:**

**All Authors**: No reported disclosures

